# Asymmetric reproductive isolation between terminal forms of the salamander ring species *Ensatina eschscholtzii *revealed by fine-scale genetic analysis of a hybrid zone

**DOI:** 10.1186/1471-2148-11-245

**Published:** 2011-08-22

**Authors:** Thomas J Devitt, Stuart JE Baird, Craig Moritz

**Affiliations:** 1 Museum of Vertebrate Zoology and Department of Integrative Biology, 3101 Valley Life Sciences Building, University of California, Berkeley, CA, USA 94720-3160; 2 CIBIO, University of Porto, Campus Agrário de Vairão, Rua Padre Armando Quintas 7, 4485-661 Vairão, Portugal; 3 Departamento de Zoología, Instituto de Biología, Universidad Nacional Autónoma de México, Circuito exterior s/n, Ciudad Universitaria, Copilco, Coyoacán, A.P. 70-153/70-233 México, Distrito Federal. C.P. 04510

## Abstract

**Background:**

Ring species, exemplified by salamanders of the *Ensatina eschscholtzii *complex, represent a special window into the speciation process because they allow the history of species formation to be traced back in time through the geographically differentiated forms connecting the two terminal forms of the ring. Of particular interest is the nature and extent of reproductive isolation between the geographically terminal forms, in this case *E. e. eschscholtzii *and *E. e. klauberi*. Previous studies have documented infrequent hybridization at the end of the ring. Here, we report the first fine-scale genetic analysis of a hybrid zone between the terminal forms in southern California using individual-based Bayesian analyses of multilocus genetic data to estimate levels and direction of hybridization and maximum-likelihood analysis of linkage disequilibrium and cline shape to make inferences about migration and selection in the hybrid zone.

**Results:**

The center of the hybrid zone has a high proportion of hybrids, about half of which were classified as F1s. Clines are narrow with respect to dispersal, and there are significant deviations from Hardy-Weinberg equilibrium as well as nonrandom associations (linkage disequilibria) between alleles characteristic of each parental type. There is cytonuclear discordance, both in terms of introgression and the geographic position of mitochondrial versus nuclear clines. Genetic disequilibrium is concentrated on the *eschscholtzii *side of the zone. Nearly all hybrids possess *klauberi *mtDNA, indicating that most hybrids are formed from female *klauberi *mating with male *eschscholtzii *or male hybrids (but not vice versa).

**Conclusions:**

Our results are consistent with a tension zone trapped at an ecotone, with gene combinations characteristic of *klauberi *showing up on the *eschscholtzii *side of the zone due to asymmetric hybridization. We suggest that the observed asymmetry is best explained by increased discriminatory power of *eschscholtzii *females, or asymmetric postzygotic isolation. The relatively high frequency of hybrids, particularly F1s, contrasts with other contacts between the terminal forms, and with other contacts between other divergent *Ensatina *lineages, highlighting the diverse outcomes of secondary contact within a single species complex.

## Background

**Figure 1:**
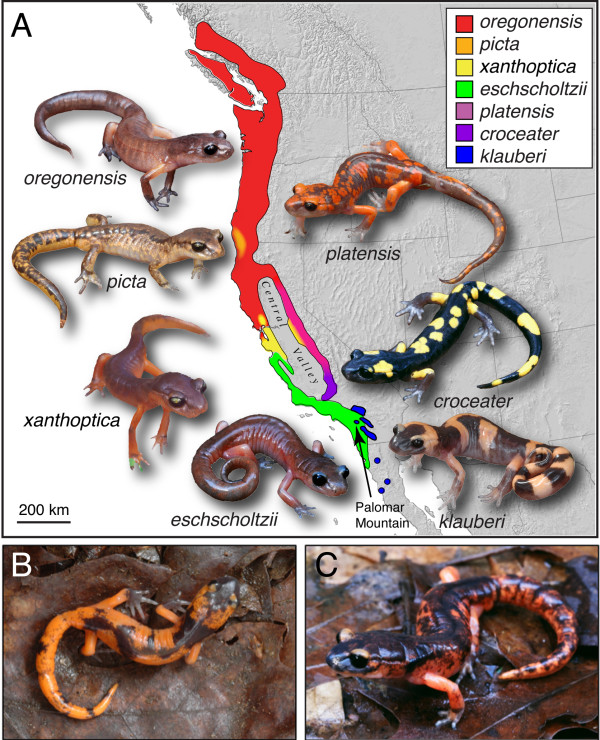
**A) Range of the *Ensatina eschscholtzii *complex and location of the hybrid zone described here (arrow); B-C) hybrid individuals showing intermediate color pattern**.

Theoretical models predict that speciation can occur among continuously distributed populations isolated by distance alone, particularly when subject to divergent ecological selection [1-3]. Ring species -- cases where two sympatric forms are connected by a chain of intergrading populations encircling a central geographic barrier [4-7] -- present the ideal opportunity to test this prediction [8,9], especially when the interacting lineages are ecologically divergent. Of particular interest in such cases is the nature and extent of reproductive isolation between the terminal forms where they are sympatric [1,10,11]. The *Ensatina eschscholtzii *plethodontid salamander complex of western North America is a famous example of a ring species [5,12-15]. These salamanders inhabit mesic, forested environments in Pacific western North America, and in California form a geographic ring around the arid Central Valley (Figure 1). In his detailed analysis of geographic variation and speciation in *Ensatina*, Stebbins [14] hypothesized that the complex originated in northern California or southern Oregon, where an ancestral range forked into two fronts that expanded southward around the arid Central Valley of California along two separate paths, one along the relatively low-elevation coast ranges, the other inland along the western slopes of the higher-elevation Sierra Nevada. Eventually, the two lineages came back into contact in southern California, creating a ring encircling the Central Valley (Figure 1). Stebbins [14] described broad (up to 150 km) intergrade zones where neighboring subspecies met, except where the two ends meet in southern California. Decades of genetic work by D. B. Wake and colleagues using allozymes and mitochondrial DNA have uncovered remarkably high, geographically structured genetic diversity within *Ensatina*, suggesting the biogeographic history of *Ensatina *is much more complex, having featured periods of geographic isolation and multiple instances of secondary contact [13,15-18]. However, these studies are consistent with Stebbins' general biogeographic hypothesis in that 1) genetic distances are higher between the geographically terminal populations than most adjacent populations [13,18], and 2) within subspecies, there is a positive correlation between genetic and geographic distance [16]. Studies of hybridization and introgression within and among subspecies have identified broad zones of introgression among ecologically similar lineages (i.e., within subspecies), but abrupt transitions between ecologically divergent subspecies [13,19].

The conclusion that the two geographically terminal subspecies, *eschscholtzii *and *klauberi*, are reproductively isolated is central to the ring species interpretation, and also to unresolved debates about species boundaries in this complex [20-23]. Although the sympatry of the terminal forms was not yet known to Stebbins in 1949, it was later discovered that these forms are sympatric in four geographically isolated contact zones in southern California [24-27]. Brown [24] examined color pattern and blood serum proteins from three of these contact zones, including a region on Palomar Mountain covering approximately 10 square miles where several narrow zones of sympatry were found with little evidence of introgression [24]. Marked differences in the habitats occupied by *klauberi *and *eschscholtzii *were noted in the Palomar region, with *eschscholtzii *occupying oak-chaparral associations below ~1,370 m and *klauberi *inhabiting pine-oak-cedar forest above this elevation [24]. Hybridization occurred in an ecotone between these two habitat types between ~1,220-1,370 m [24]. Wake et al. [28] typed samples of individuals from all four contact zones at 26 allozyme loci, finding evidence of hybridization (albeit infrequent) at all but the southernmost contact in the Cuyamaca Mountains (see also [13]).

Although these studies provided the first evidence for hybridization between *klauberi *and *eschscholtzii*, individuals were sampled at a relatively broad geographic scale, and analyses were limited to identification and classification of hybrid individuals. The present study extends previous work on hybridization at the end of the ring in three ways. First, the geographic scale of this study is much finer than previous work, an important consideration given that hybrid zones may vary in structure (i.e., clinal vs. mosaic) at different sampling resolutions [29-31]. In general, sampling should be undertaken at the scale at which individuals meet, mate, and produce offspring (tens of meters in *Ensatina*; [32,33]). Second, although habitat isolation has been implicated as a strong barrier to gene flow between sympatric populations of *Ensatina *[19,24,28], our study is the first to examine whether habitat-genotype associations exist and to formally test the hypothesis that habitat type is structuring the hybrid zone [24]. Finally, this study is the first to use maximum-likelihood analysis of multilocus clines and linkage disequilibrium to make inferences about selection in a hybrid zone at the end of the ring.

A solid theoretical framework exists for studying the evolution and maintenance of clines [34-37]. After any initial contact between differentiated populations, clines in allele frequencies will exist, the scale of which depends on the dispersal scale of the organism, the strength of selection and time since contact, and the shape of which will be determined by asymmetric and epistatic effects [2,38]. If there is neutral mixing, steep gradients in allele frequencies will decay over time resulting in wide, shallow clines; conversely, if there is a strong barrier to gene flow because of selection against hybrids and/or assortative mating, steep clines may be maintained [2,10,39]. Selection may act against hybrids independent of geographic location, or along environmental gradients where local adaptation favors one parental form in one habitat, and the other in another [34-36]. In the latter scenario, migrants dispersing into the neighboring habitat keep the taxa mixed and prevent further local adaptation. All of these models assume that dispersal is random with respect to phenotype and the environment. However, it is possible that an individual's decision about whether and where to disperse depends on its fitness in a given environment, with different genotypes moving into preferred habitat patches where they are locally adapted [40,41]. It seems reasonable that such fitness-dependent dispersal may result in fine-scale genotype-habitat associations [42]; when such associations exist, simple hybrid zone models may not effectively describe spatial variation in allele frequencies [43,44], but can be extended to take such associations into account [45]. In addition, even when selection against hybrids/hybridization is not tied to the environment, tension zones resulting from endogenous selection will tend to seek out geographic barriers to gene flow and/or habitat suitability troughs in the environment, where they will become trapped [38]. The interaction of endogenous and exogenous factors where tension zones come to rest may further steepen clines. For these reasons, incorporating information on local environmental variation is important in understanding the balance between local adaptation and dispersal and its effect on hybrid zone structure.

Here, we present a detailed genetic analysis of a hybrid zone on Palomar Mountain between the geographically terminal forms of the *Ensatina *complex. Our goal is to analyze the barrier to gene flow between these sympatric forms to provide insight into the nature and extent of reproductive isolation between the terminal forms of a ring species. We use two complementary analytical approaches. First, we use individual-based Bayesian methods for identifying and classifying hybrids using multilocus genetic data to estimate levels and direction of hybridization [46,47]. We then use maximum-likelihood analysis of linkage disequilibrium and cline shape to make inferences about dispersal and selection in the hybrid zone [48,49]. We test for genotype-habitat associations, and whether including this variation improves the fit of clines [cf. 45]. If a strong barrier to gene flow exists between the hybridizing taxa, we would expect to see: 1) concordant clines among independent loci that are narrow with respect to dispersal; 2) deviations from Hardy-Weinberg equilibrium at diagnostic loci due to nonrandom mating and/or selection against hybrids; and 3) nonrandom associations (linkage disequilibria) between alleles characteristic of each parental type, due predominantly to dispersal of parental individuals into the hybrid zone [10,48-50]. By examining patterns of cytonuclear disequilibria, we provide insight into possible mechanisms of selection and patterns of mating in the hybrid zone [51] that may be important for maintaining species boundaries. Finally, we discuss the evolutionary implications of introgression and reproductive isolation at the end of the ring for divergence within the *Ensatina *complex as a whole.

## Methods

### Sampling

**Figure 2:**
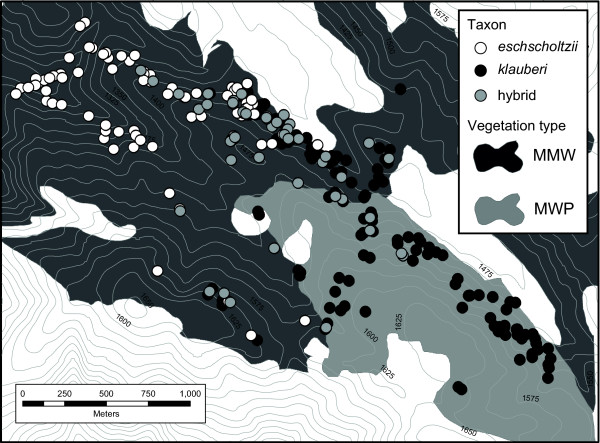
**Distribution of *klauberi*, *eschscholtzii*, and hybrid individuals across the contact zone (based on phenotype and genotype)**. Suitable habitat is represented by gray polygons, where MMW = Mixed Montane Woodland and MWP = Mixed Woodland with Bigcone Douglas fir (*Pseudotsuga macrocarpa*). Vegetation map modified from [70].

The study site is located on Palomar Mountain, San Diego County, California (Figure 1). Suitable habitat consists of mixed montane woodland and montane riparian forest dominated by Incense Cedar (*Calocedrus decurrens*), White Fir (*Abies concolor*), Black Oak (*Quercus kelloggi*), Coast Live Oak (*Quercus agrifolia*), Bigcone Douglas Fir (*Pseudotsuga macrocarpa*), and Canyon Live Oak (*Quercus chrysolepis*). The area sampled is approximately 3.5 km × 1.75 km. The sampling distribution primarily follows a northeast-facing slope and boulder-filled creek adjacent to an open treeless meadow of unsuitable habitat (Figure 2). Salamanders were captured using visual surveys of natural cover objects during the day and night driving. All individuals that were encountered were sampled, and latitude, longitude, and elevation (with error estimates < 10 m) were recorded at the point of capture using a GPS (Additional file 1). Three hundred and thirty-five salamanders were sampled over a three-year period from January-April of 2005-7. Individuals were classified based on diagnostic color pattern as *klauberi*, *eschscholtzii*, or hybrids (Additional file 1). Hybrids are readily identified by their aberrant color pattern (Figure 1). Most individuals were sampled non-lethally by removing a piece of the tail tip to allow for future long-term monitoring of the nature of this zone; a subset of individuals were euthanized and preserved as voucher specimens and deposited in the Museum of Vertebrate Zoology (MVZ), University of California, Berkeley. Tissue samples collected in the field were stored in 95% ethanol or propylene glycol and later frozen at -80°C in the lab. Individuals that were not collected were photographed and marked using subcutaneous alphanumeric tags (Northwest Marine Technology, Shaw Island, WA) and returned to their point of capture within 24 hours. All research procedures using live animals were conducted in accordance with the University of California, Berkeley's institutional animal care and use committee protocol (R278-0410) issued to CM.

### Molecular markers

DNA was extracted from tissues (liver or tail-tip) using Qiagen DNeasy tissue kits following the manufacturer's protocol (Qiagen, Valencia, CA). Using PCR, we amplified three nuclear protein-coding loci (CXCR4, SLC8A3, RAG1) and one mitochondrial locus (ND4) for all 335 individuals. PCRs consisted of 40 cycles of 94°C for 30 s, Ta°C for 45 s, and 72°C for 60 s, with locus specific annealing temperatures (Table 1). Primer sequences are based on primers published elsewhere [52-55]. PCR products were purified using ethanol following standard methods. The mitochondrial PCR product was used in a PCR-RFLP assay with the *Hind*III restriction endonuclease following the manufacturer's protocol (New England Biolabs, Ipswich, MA). The enzyme recognizes a single restriction site in individuals with *klauberi *mitochondrial DNA, resulting in a double-band profile for *klauberi *mtDNA and a single-band profile for *eschscholtzii *mtDNA. Restriction fragments were visualized on standard agarose gels and scored. Autosomal loci were sequenced in the forward direction only for most individuals. Purified templates were sequenced using dye-labeled dideoxy terminator cycle sequencing. DNA sequences were edited and aligned using Geneious Pro v5.3 [56]. Raw DNA sequence alignments are provided in additional files 2, 3 and 4. Invariant sites and ambiguous positions were removed from the alignments. We resolved haplotypes using PHASE [57,58] after first using SeqPHASE [59] to convert between FASTA and PHASE files.

**Table 1:** PCR annealing temperatures, sequence length, and primer sequences

Locus	Annealing **temp**.	Length/ # variable sites (bp)	PCR primer	Primer sequence (5'-3')
ND4	48°C	RFLP	ND4	CACCTATGACTACCAAAAGCTCATGTAGAAGC
			LEU	ACCACGTTTAGGTTCATTTTCATTAC
CXCR4	55°C	116/4	CXCR4F2	TGGTCTGTGGATGCTGTCAT
			CXCR4R	TGCAGTAGCAGATCAAGATGA
SLC8A3	58°C	342/6	SLC8A3F	CATTCGGGTCTGGAATGAAA
			SLC8A3R	ACACCACCATCCCCTCTGTA
RAG1	56°C	359/7	Amp F1	ACAGGATATGATGARAAGCTTGT
			RAG1R	TTRGATGTGTAGAGCCAGTGGTGYTT

### Identification and classification of hybrids

Structure [47,60,61] and NewHybrids [46,62] were used to identify and classify hybrids using the multilocus sequence data. In the context of a two-population hybrid zone, Structure jointly assigns individuals probabilistically to the two parental populations [47,60,61], while NewHybrids computes the posterior probability that an individual belongs to distinct genotype frequency classes (e.g., parentals, F1s, F2s, and backcrosses) arising from early generation matings between two species [46,62]. We coded each unique haplotype as a different allele (Additional file 5). In Structure, we used the admixture model and assumed two populations with independent allele frequencies (λ = 1). We ran 100,000 sweeps of five chains after a burn-in of 50,000 sweeps and checked for convergence by comparing the estimated membership coefficient (*Q*) for each individual across the 5 runs. For NewHybrids, we used the default genotype categories for first- and second-generations of crossing and ran 100,000 sweeps of five chains started from overdispersed starting values after a burn-in period of 50,000 sweeps following the software author's recommendation. Uniform priors were used for the mixing proportions and allele frequencies. To check for convergence, we visually inspected *P*(*z*) values from the different runs which were then averaged across the 5 runs. A threshold hybrid index (*Q*-value) was calculated to classify individuals as "hybrids" or "pure parentals" in Structure. For three diagnostic loci (six alleles), there are seven categories (i.e., an individual may contain 0, 1, 2, 3, 4, 5, or 6 alleles from taxon 1) and thus "hybrids" were defined as any individual with a *Q*-value between 0.1 [i.e., (0+1/7)/2] and 0.9 [i.e., (1+6/7)/2], while any individual with a *Q*-value < 0.1 or > 0.9 was considered to be a pure parental. The same threshold was used to distinguish hybrids from pure parentals for the NewHybrids analysis, with posterior probability values summed across hybrid classes for an individual [46,62]. Information about the taxon of origin of individuals was not used for either analysis.

### Cline analysis

Phased haplotypes were used to identify allelic states associated with each source taxon (i.e., "*eschscholtzii*" and "*klauberi*" alleles) for cline analysis. All loci were diagnostic for one parental form or the other, allowing nearly all haplotypes to be unambiguously assigned. Two haplotypes found in four individuals for one of the loci (SLC8A3) could not be unambiguously assigned and were excluded from the cline analysis. We assumed that allele frequencies at individual loci did not change significantly over the three-year sampling period. Individuals were not pooled into discrete samples for cline fitting.

Sampling sites were collapsed onto a one-dimensional transect using the Pooled Adjacent Violators Algorithm (PAVA) [63], a method for finding the maximum-likelihood (ML) monotonic cline over a set of observations [64]. The advantage of the PAVA method is that it doesn't depend on a particular cline model; it assumes only a monotonic increase or decrease in allele frequencies across sampling sites [64]. Because the path of the contact front is assumed to be linear in the PAVA method, the best-fit axis of the transect orientation through sampled individuals was estimated using a one-dimensional cline fitting with a straight centerline. The best-fit axis of orientation equated to a heading of 207° (support limits 200°, 211°). There was no evidence for different orientation among different loci. Monotonic clines and their likelihood profiles were estimated using a routine written in Mathematica [65] by SJEB for Analyse 2.0beta. The width of a cline is usually defined as the inverse of the maximum slope [37]. However, because it is not clear how best to estimate the maximum slope from monotonic clines, we estimated cline widths by fitting parametric sigmoid clines using Analyse 1.3 [66]. A sigmoid cline in allele frequencies (*p*) can be modeled by a tanh function of its width and center, such that

p=(1+tanh[2(x−c)/w])/2

where *c *is the cline center, *w *is the cline width and (*x *- *c*) is the distance from the cline center [48]. We fit sigmoid clines and did not explore more complex stepped cline models because doing so only seems justified when there is sufficient sampling in the tails of the cline [38]. Parameter estimates are given as maximum-likelihood estimates, along with two-unit support limits analogous to 95% confidence intervals [67].

Cline shape concordance among loci was assessed using Barton's concordance analysis [48,68]. In Barton's approach, a hybrid index (*HI*) is calculated for each individual and scaled from 0 to 1, expressed as the proportion of alleles derived from one of the two parental types (in this case, *klauberi*). This set of alleles can be further partitioned into subsets of alleles coming from different loci (e.g., the individual's alleles from locus 1) and a *HI *calculated for each subset, resulting in individual × locus *HI*s [68]. If all loci introgress equally, the expectation for an individual × locus *HI *is the same as for an individual's *HI*. Thus, under the expectation for equal introgression across loci, the plot of individual × locus *HI*s against individual *HI*s will fall on the diagonal (i.e., *x = y*). Deviations from this equal-introgression expectation can be expressed as

$$HI_{loc}=HI+H_{e}\left[\alpha +\beta \left(2HI-1\right)\right]$$

where the locus expectation can deviate from *x = y *as a function of the expected heterozygosity, *He *= 2*HI*(1 - *HI*). The α parameter (directionality) shifts introgression toward one or the other genome (in this case, toward *eschscholtzii*) and the β parameter (abruptness) allows the introgressive change at a locus to be more or less abrupt than the equal-introgression expectation [68].

Barton's concordance method [48] is an individual-based method independent of geographic information. We also fitted geographic clines to each locus separately and assessed cline coincidence using the likelihood method described by Phillips et al. [50]. The likelihood surface of each locus was explored stepwise along axes for both center position (*c*) and width (*w*) while allowing other parameters to vary at each point [50]. Likelihood profiles [69] were constructed for both *c *and *w *and summed over all loci, resulting in a log-likelihood profile for the ML shared center or width [50]. The shared ML estimate was compared to the sum of noncoincident profile ML estimates using a likelihood ratio test [69]. Twice the difference in log likelihood (*G *= 2ΔLL) between the two models under comparison is significant at level α if *G *= 2ΔLL > χ^2^_df, α _with the degrees of freedom equal to the difference in the number of parameters between the two models [48,49].

### Testing for genotype-habitat associations

We tested whether hybrids and pure parentals show differences in elevation and vegetation type to see if a simple clinal hybrid zone model was appropriate for describing spatial variation in allele frequency. If significant genotype-habitat associations exist, a simple clinal model may be a poor description of the observations [43] relative to a clinal model that takes such associations into account [45]. We used elevation estimates taken at the point of capture for each individual and vegetation data from a floristic study of Palomar Mountain State Park [70]. Individuals were classified as coming from one of two vegetation series, Mixed Montane Woodland (MMW) or Montane Woodland with *Pseudotsuga macrocarpa *(MWP) (Figure twenty-eight in [70]). These series intergrade [70] and do not have sharply defined borders in and around the hybrid zone, but there is a discernible transition from MMW in the northwestern, lower-elevation portion of the transect to MWP in the southeastern, higher-elevation portion of the transect (Figure 2). Statistical analyses were performed using SPSS Statistics 17.0 (IBM).

To examine whether genotype-habitat associations contributed to the observed spatial structure of the hybrid zone, we compared a model incorporating variation in habitat and elevation (the "habitat-and-cline" model) to one assuming no genotype-habitat associations (the "cline only" model" model) using likelihood-ratio tests [cf. 45], accepting the more complex (i.e., more parameter rich) model only when justified by a significant increase in the likelihood of the observations.

### Estimating linkage disequilibria

Average pairwise linkage disequilibrium (*D*) was estimated in sets of sampled genotypes using the likelihood approach implemented in Analyse 1.3 [66]. Sets of genotypes were formed in a window sliding across the most likely orientation of the hybrid zone using a window size of 200 meters moved in 100 meter increments along the cline using a routine written in Mathematica [65] and implemented in Analyse 2.0beta. *D *is a measure of the statistical association of allelic states across loci, which ranges from -0.25 through zero to 0.25. When considering a single diploid locus (rather than two haploid loci), the analog of *D *is heterozygote deficit [71]. We calculated: 1) within locus disequilibrium (the ML estimate of heterozygote deficit over the three diploid nuclear loci); 2) between nuclear loci disequilibrium (the ML estimate of pairwise *D *over the three possible pairs of the nuclear loci); and 3) cytonuclear disequilibrium (the ML estimate of pairwise *D *over the three possible pairs of nuclear/mt markers).

## Results

### Hybrid zone genotypes

**Figure 3:**
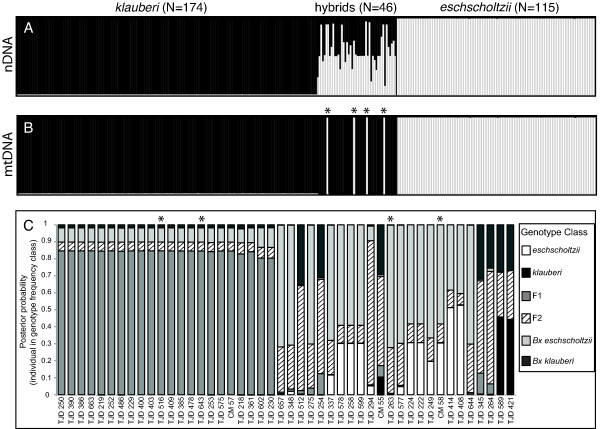
**Identification and classification of hybrids**. A) Structure results showing proportion of membership from each parental population for all 335 individuals; B) MtDNA haplotypes for all individuals; C) NewHybrids classification of the 46 hybrids classified by genotype frequency class. The four individuals with asterisks in (B) and (C) represent hybrids with *eschscholtzii *mtDNA; all other hybrids possess *klauberi *mtDNA.

There is a clear transition at all loci from *eschscholtzii *alleles in the northwest to *klauberi *alleles in the southeast. Hybrids are concentrated near the estimated consensus center of the hybrid zone. Initial identification of individuals based on color pattern as *eschscholtzii*, *klauberi*, or hybrid was generally consistent with genetic classification based on Structure and NewHybrids (Additional File 1). Forty-six individuals initially identified as hybrids based on color pattern were classified as such by Structure with a *Q*-value between 0.1 and 0.9, and by NewHybrids with posterior probability > 0.8 (Figure 3A). Of the 46 hybrid individuals, 42 possessed *klauberi *mtDNA (Figure 3B). Twenty-two of the 46 hybrid individuals were classified as F1s with posterior probabilities > 0.8 (Figure 3C). Where individuals were estimated to be F2s or backcrosses, the estimates were not well-supported (Figure 3C), though these individuals appear to contain more *eschscholtzii *alleles. Of the four hybrids with *eschscholtzii *mtDNA, two were classified as F1s (*pp *> 0.85; Figure 3C).

### Genotype-habitat associations

Parental forms are associated with different vegetation series in the contact zone (Chi-square test, *p *< 0.001). *Eschscholtzii *are found almost exclusively in the MMW series along with > 80% of the hybrids, while *klauberi *individuals are found at roughly equal frequencies in the two vegetation series. *Eschscholtzii *occupies a broader, but lower elevational range (1200-1601 m; mean 1360 m) than *klauberi *(1297-1694 m; mean 1470 m) and hybrids (1342-1601 m; mean 1445 m) (One-way ANOVA, Tukey's HSD, *p *< 0.001). Modification of the underlying clines according to vegetation type [cf. 45] gave no significant improvement in cline fit, and modification according to elevation provided only a slight improvement (results not shown).

### Cline shape and concordance

**Figure 4:**
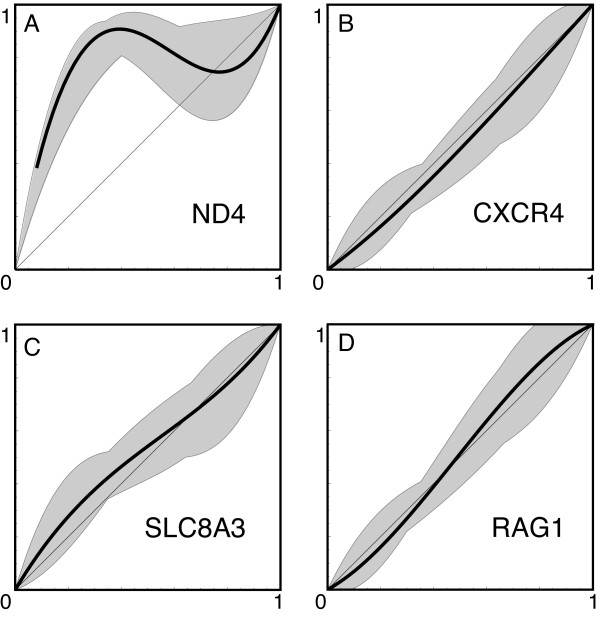
**Comparison of mitochondrial (A) and nuclear (B-D) cline concordance using Barton's concordance analysis**. Plots show the individual per-locus hybrid index plotted against the hybrid index calculated over all loci. The equal-introgression expectation lies on the diagonal. Per locus fits can deviate toward either genetic background (α) and by having more or less abrupt change (β) (see text). The nuclear clines are concordant with each other (and the consensus nuclear cline), while the mitochondrial cline is not; individuals are more likely to have *klauberi *mtDNA than expected under equal introgression across loci (α = 0.753).

**Figure 5:**
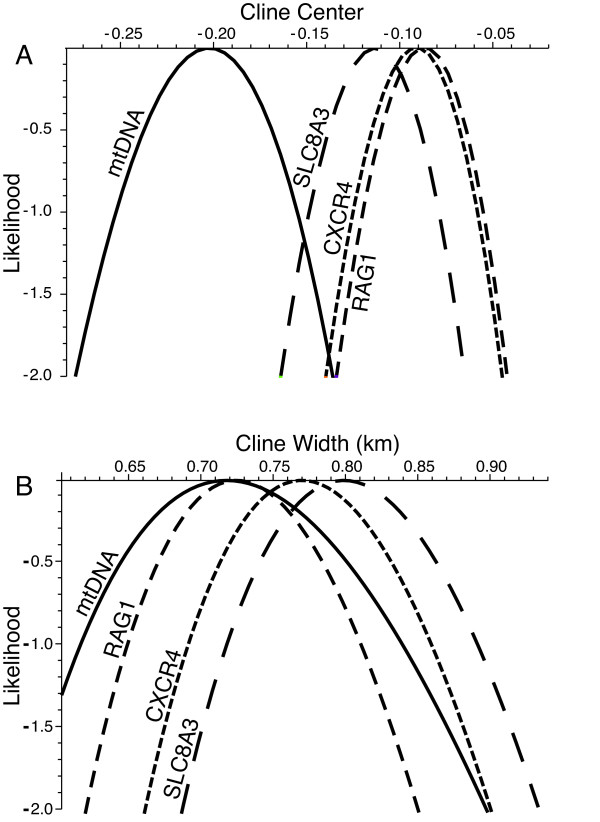
**Maximum likelihood profiles for the centers (A) and widths (B) of clines**. Cline centers for the nuclear loci are concordant with each other, while the mitochondrial cline is shifted significantly to the west (A). There is no significant difference in cline widths (B).

Cline width and center estimates and the α and β components of Barton's [48] concordance analysis are provided in Table 2. Both mitochondrial and nuclear loci change over approximately the same distance (cline widths of 718-799 m; Table 2). The three nuclear loci were concordant (Figure 4B-D). The mitochondrial locus however, was not concordant with the nuclear loci (Figure 4A), showing a shift toward the range of *eschscholtzii *by approximately 15% of the consensus cline width (approximately 100 m). Maximum-likelihood profiles for the centers and widths of clines are shown in Figure 5. Likelihood-ratio tests showed no significant difference in cline width when all four loci were considered separately versus when they were considered together.

**Table 2:** Maximum-likelihood estimates (with lower, upper support limits) of cline centers and widths along with the α and β components of Barton's concordance analysis [48,68]

Locus	Center	Width (km)	α	β
*All loci*	- 0.112 (- 0.138, - 0.087)	0.764 (0.704, 0.830)	--	--
ND4	- 0.202 (- 0.274, - 0.136)	0.718 (0.580, 0.898)	0.753	- 1.522
*All nuclear loci*	- 0.097 (- 0.125, - 0.070)	0.765 (0.700, 0.838)	--	--
CXCR4	- 0.091 (- 0.140, - 0.045)	0.770 (0.661, 0.901)	- 0.091	0.040
SLC8A3	- 0.113 (- 0.164, - 0.065)	0.799 (0.687, 0.934)	0.084	- 0.258
RAG1	- 0.087 (- 0.134, - 0.042)	0.725 (0.620, 0.851)	0.026	0.248

Center and width units are in kilometers and centers are measured with the gnomonic projection focus at zero.

### Linkage disequilibria

**Figure 6:**
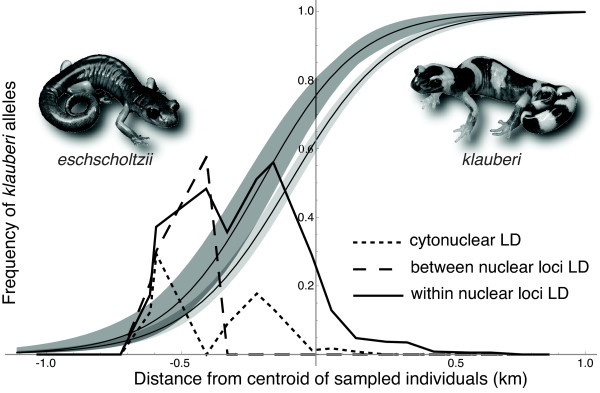
**Mitochondrial (dark gray) versus consensus nuclear geographic clines (light gray) along with support envelopes**. Genetic disequilibria (*D*) estimates (multiplied by four for visualization purposes) are superimposed on the clines. The short-dashed line represents cytonuclear disequilibrium (MLE pairwise *D *over 3 possible pairs of nuclear/mt loci), the long-dashed line represents between nuclear loci disequilibrium (MLE pairwise *D *over 3 possible pairs of the nuclear markers), and the solid line represents within locus disequilibrium (MLE of heterozygote deficit over the three nuclear loci).

Results of the sliding-window analysis of linkage disequilibria are shown in Figure 6. Genetic disequilibrium (including heterozygote deficit and cytonuclear disequilibrium) is concentrated on the *eschscholtzii *side of the hybrid zone, where within and between nuclear loci estimates of *D *reach maximum values of approximately 0.15. Cytonuclear disequilibrium is found exclusively on the *eschscholtzii *side of the zone.

## Discussion

Earlier work based on allozymes has shown that hybridization is infrequent, or even absent in at least one area of sympatry at the end of the *Ensatina *ring [27,28]. Our fine-scale analysis revealed a much higher frequency of hybridization compared to previous estimates for Palomar Mountain as well as other contacts between *eschscholtzii *and *klauberi *based on allozymes and morphology [27,28]. Pure parentals and F1s dominated the sample. The hybrid zone is narrow (cline width estimates of 718-799 m) with respect to per generation dispersal estimates (maximum ~120 m; [32]). Although significant genotype-habitat associations exist, modification of the underlying clines according to vegetation type did not improve the fit of clines, and modification by elevation provided only a minor improvement. The predominance of genetic disequilibrium (including heterozygote deficit and cytonuclear disequilibrium) on the *eschscholtzii *side of the hybrid zone, coupled with the fact that nearly all hybrids have *klauberi *mtDNA suggests that either: 1) the hybrid zone may be moving (toward the range of *eschscholtzii*) and/or 2) the zone is static, but gene flow is asymmetric (from *klauberi *to *eschscholtzii*). Without long-term data on the spatiotemporal dynamics of the zone, these hypotheses are difficult to disentangle [10]. Barton and Hewitt [10] argued that most hybrid zones are clines maintained by a balance between dispersal and selection against hybrids ("tension zones" [72]), and can move from place to place because they are not maintained by local environmental conditions. Tension zone movement may be caused by differences in fitness, density, dispersal, or hybridization asymmetry between parental forms [2,10,73,74]. For this reason, although they are not maintained by the local environment, tension zones will move toward and become associated with barriers to gene exchange, including environmental factors that reduce density or dispersal [10]. Our observations are consistent with a tension zone trapped at an ecotone, with gene combinations characteristic of *klauberi *showing up on the *eschscholtzii *side of the zone due to asymmetric hybridization, giving a strong signal of genetic disequilibria. Given that nearly all of the hybrids possess *klauberi *mtDNA, either: 1) hybridization between *eschscholtzii *and *klauberi *is (mostly) unidirectional, with F1 hybrids formed from female *klauberi *mating with male *eschscholtzii *(but not vice versa), implying asymmetric prezygotic isolation, or 2) hybridization is reciprocal, but offspring resulting from female *eschscholtzii *mating with male *klauberi *(or male hybrids) are inviable (implying asymmetric postzygotic isolation [75]). Hybrids often possess the mitochondrial DNA of only one of two parental species in nature [76-78], a pattern predicted in species with female-choice when females of a rare species are unable to find conspecific males because they are scarce, and eventually accept matings with heterospecific males of a more common species [79]. However, the two taxa appear to be equally abundant in zones of overlap at Palomar Mountain, suggesting that factors other than rarity of conspecific *klauberi *males are responsible for the disproportionate percentage of hybrids with *klauberi *mtDNA. The presence of four hybrid individuals with *eschscholtzii *mtDNA suggests matings between female *eschscholtzii *and male *klauberi *(or male hybrids) are possible, but appear to be rare, or that the offspring usually do not survive. Like many animals, female *Ensatina *are the choosier sex because they invest relatively more than males in reproduction, and are courted by promiscuous males, both of their own and closely related species [1,80]. If there are costs associated with heterospecific matings (e.g., because hybrids are less fit, or, because these matings produce fewer offspring), there may be strong selection acting on female mating preferences toward increased ability to discriminate between conspecific and heterospecific males [81-83]. This in turn could drive indirect selection on males to track female mating preferences [84].

It is instructive to compare the nature of hybridization between the terminal forms to that between another pair of morphologically and genetically distinct coastal and inland lineages of *Ensatina *-- *xanthoptica *and *platensis*, respectively -- in the foothills of the central Sierra Nevada [19,24,28]. These morphological analogs of *eschscholtzii *and *klauberi *are thought to have come into contact at some point during the Pleistocene when climate in California was cooler and wetter, allowing *xanthoptica *to cross the now arid Central Valley and invade the Sierras from the San Francisco Bay area [14]. Hybrids are abundant in the Sierran contact zones, but few, if any, F1s have been detected [19], in contrast to our results. Alexandrino et al. [19] suggested that reduced opportunities for heterospecific encounters due to habitat preference and/or stronger selection against F1s compared to later generation hybrids could explain this pattern. They estimated cline widths comparable to, or wider than those observed here (730-2000 m), and inferred strong selection against hybridization (46-75%). For comparison, selection against hybridization has been estimated at 32% for distinct lineages of lizards of the *Sceloporus grammicus *complex [85,86] and 17-22% for the toads *Bombina bombina *and *B. variegata *[48,49]. Although it is tempting to try and estimate selection against hybridization in the contact zone studied here, because the hybrid zone is dynamic and/or asymmetric, we cannot sensibly do so under a standard tension zone model, which assumes a symmetric, static hybrid zone. Nonetheless, it is clear that overall selection against hybrids/hybridization is strong in both cases, yet the two contact zones differ markedly in the frequency of different hybrid genotype frequency classes.

## Conclusions

Geographic variation in hybridization frequency among the four contact zones at the end of the ring [27,28] suggests that reproductive isolation may not be uniform across contact zones, perhaps due to differences in local ecological conditions that limit the opportunity for hybridization [see 87], and/or different outcomes of reinforcement [88,89] driven by selection against hybrids owing to the spatial structure of the hybrid zone [43,90,91]. This variation provides a rare opportunity to investigate variation in the strength of reproductive isolating barriers within a single species pair in nature, while controlling for among-taxa variation in age, degree of differentiation, strength of postzygotic isolation, etc. [see 1, 87, 92].

The narrow width, shape, and overall concordance of clines for presumably unlinked loci suggest a strong barrier to gene flow between *eschscholtzii *and *klauberi *on Palomar Mountain. Introgression of neutral alleles will be delayed, but favorable alleles could quickly cross if prezygotic isolation is weak or absent [93,94]. The particular markers themselves probably do not have a direct effect on fitness, but rather they are in linkage disequilibrium with other loci that are under selection. If the amount of linkage disequilibrium that connects selection against hybrids (or hybridization) with an evolving prezygotic isolating mechanism is sufficiently high, then premating isolation may evolve through reinforcement [95]. Future work incorporating information about patterns of mating and gamete utilization will be critical for understanding the role of selection in generating and maintaining species boundaries at the end of the ring [83,96,97].

## Authors' contributions

TJD designed the study, collected the material and data, performed the Bayesian analyses, interpreted results, and wrote the manuscript. SJEB performed the cline and genetic disequilibria analyses, interpreted results, and provided extensive feedback that greatly improved the manuscript. CM provided logistical support and significant intellectual contributions that improved the study and final manuscript. All authors read and approved the final manuscript.

## Supplementary Material

Additional file 1 ***Sampled individuals***. For each individual, geographic coordinates (latitude and longitude), elevation, vegetation series, Structure and NewHybrids classification, and alleles for each marker (E = *eschscholtzii*, K = *klauberi*) are given. Missing data is represented by -9. Samples without a catalog number represent tissues that were depleted during DNA extraction. Click here for file

Additional file 2 **Sequence alignment for CXCR4 in FASTA format**. Click here for file

Additional file 3 **Sequence alignment for RAG1 in FASTA format**. Click here for file

Additional file 4 **Sequence alignment for SLC8A3 in FASTA format**. Click here for file

Additional file 5 ***Phased haplotypes***. For each individual, variable nucleotide positions (where A = 1, C = 2, G = 3, T = 4) and coded haplotypes used in Structure and NewHybrids analyses are given. Missing data is represented by -9. Click here for file
